# Hospital Meetings

**Published:** 1904-12-17

**Authors:** 


### HOSPITAL MEETINGS.

### THE LONDON HOSPITAL.

The special Quarterly Court of Governors was held at the London Hospital on Wednesday in last week. The Hon. Sydney Holland presided, and among those present were Mr. W. Howlett, Mr. W. Moss, Mr. Clogg, Mr. C. J. Bone, the Rev. S. Vatcher, Mr. J. A. Evans, Mr. G. V. Morrice, Mr. M. Scott, Mr. E H. Pearce, Mr. C. Hart, Mr. R. Biscoe, Mr. F. H. Barnes. The minutes of the last meeting were read, and agreed by all present to be correct.

The Chairman then went on to read the report of the last quarter. In it he stated that the rebuilding and structural alterations were nearing completion. Since the last meeting a west wing—the top floor of which contains the new Hebrew wards, and the basement the new coroner's court to be opened at the end of the year—was opened on November 14 by Mr. Leopold Rothschild. The new nurses' home had been roofed in. In October last the hospital was visited by the French doctors, all of whom expressed themselves delighted with what they saw. The entries of students had this term been larger than usual. Hoggart, the worst injured victim of the Baltic disaster, had been a patient. At the special request of the King, he was seen by Sir Frederick Treves. He was now doing well and had been sent to Felixstowe. While in the hospital, the Queen had sent Hoggart fruit and flowers, and her autographed photograph. The old Hebrew wards had been turned into lying-in wards. Hitherto there have been no lying-in wards in the hospital. Though the establishing of them is an expensive step, the authorities consider it to be a wise one. During this quarter the East London Hospital societies had withdrawn their support, because the London Hospital authorities had seen fit to abolish the system of letters of recommendation. The authorities considered it a sufficient recommendation if a person were sick and poor and in need of help. The chairman moved the adoption of the quarterly report, which was seconded, and carried unanimously. A short discussion ensued, as to the abolition of letters of recommendation, during which members of the General East End Tradesmen's Society in Aid of the Hospital, and of the Bethnal Green Workingmen's Society, spoke for and against the new departure. Finally, they all acquiesced to the action taken by the hospital authorities.

A vote of thanks to the chairman closed the meeting.

